# The relationships of genetic polymorphisms of the long noncoding RNA growth arrest-specific transcript 5 with uterine cervical cancer

**DOI:** 10.7150/ijms.44583

**Published:** 2020-05-18

**Authors:** Shun-Long Weng, Soo-Cheen Ng, Yueh‐Chun Lee, Yi-Hsuan Hsiao, Chun-Fang Hsu, Shun-Fa Yang, Po-Hui Wang

**Affiliations:** 1 Department of Obstetrics and Gynaecology, Hsinchu Mackay Memorial Hospital, Hsinchu City, Taiwan; 2 Department of Medicine, Mackay Medical College, New Taipei City, Taiwan; 3 Mackay Junior College of Medicine, Nursing and Management College, Taipei, Taiwan; 4 Institute of Medicine, Chung Shan Medical University, Taichung, Taiwan; 5 Department of Obstetrics and Gynecology, Chung Shan Medical University Hospital, Taichung, Taiwan; 6 School of Medicine, Chung Shan Medical University, Taichung, Taiwan; 7 Department of Radiation Oncology, Chung Shan Medical University Hospital, Taichung, Taiwan; 8 Department of Obstetrics and Gynecology, Changhua Christian Hospital, Changhua, Taiwan; 9 Department of Medical Research, Chung Shan Medical University Hospital, Taichung, Taiwan; 10 Department of Obstetrics and Gynecology, Chung Shan Medical University Hospital, Taichung, Taiwan

**Keywords:** long noncoding RNA growth arrest-specific transcript 5, genetic variants, uterine cervical cancer, precancerous lesions, 5 years survival rate

## Abstract

The purposes of the investigation were to examine the implications of long noncoding RNA growth arrest-specific transcript 5 (GAS5) in progression and clinicopathological factors of uterine cervical cancer, and patient survival in Taiwan. Genotypic distributions of two GAS5 genetic variants rs145204276 and rs55829688 were detected in 208 patients including 111 patients with invasive cancer, 97 with precancerous lesions as well as 307 control women using real-time polymerase chain reaction. It explored that patients with cervical precancerous lesion had lower rate of AGGCA deletion (Del) in both alleles (Del/Del) of GAS5 rs145204276 as compared with control women. Patients with invasive cancer did not exhibit higher rate of Del/Del. Meanwhile, there were no different genotypic distributions in rs55829688 among patients with cervical invasive cancer and those with precancerous lesions as well as control women. Moreover, cervical cancer patients with Ins (insertion, AGGCA)/Del and Del/Del (-/-) in GAS5 rs55829688 tended to have poorer hazard ratio (HR) of 5 years survival. In addition, lymph node metastasis status exerted the most significantly predictive of 5 years survival rate. Conclusively, GAS5 polymorphism rs145204276 is probably applicable to predict 5 years survival HR of cervical cancer patients. However, the mechanism elucidating the methylation status and transcription function of rs145204276 in uterine cervical cancer needs to be delineated for its unique implication in uterine cervical cancer.

## Introduction

Long noncoding RNAs (lncRNAs) growth arrest-specific transcript 5 (GAS5), firstly discovered from mouse NIH 3T3 cell by Schneider et al, is a lncRNA and situated at 1q25 locus, which is consisted of 12 exons and 11 introns 1, 2. LncRNAs are in general considered as RNA transcripts that have more than 200 nucleotides in length without amino acid coding potential. Accumulating evidence has revealed lncRNAs are concerned with various biological roles, including gene expression, cell proliferation and migratory behavior, and are involved in cancer development because of their displaying oncogene or tumor suppressor function 3, 4. LncRNA Hox transcript antisense intergenic RNA (HOTAIR) is regarded as an oncogenic lncRNA^5^, ^6^, whereas GAS5 a tumor suppressor lncRNA 7, 8.

Increasing evidence has shown that GAS5 exerts a variety of biological functions including cell proliferation, invasiveness and DNA repair in cancer 9, 10. It modulates cell cycle in various mammalian systems and displays its influence on cell proliferation via regulation of CDK6 activity 11-13. It is identified as an about 630 nucleotide lncRNA transcript and principally regarded as a tumor suppressor in human cancer. It has been demonstrated that GAS5 is down-regulated in many cancers, such as breast cancer, hepatocellular carcinoma, lung cancer and ovarian cancer 14-17. Reduced expression of GAS5 was also reported to be concerned with poor patient survival in gastric cancer patient in addition to its involvement in cancer cell proliferation 18.

Genetic polymorphism variants are referred to as allele base variation in the shared DNA sequence of genomic DNA where alternative alleles develop with a frequency of more than 5% of the human population 19, 20. When single nucleotide polymorphisms (SNPs) occur in coding sequences, they may present as synonymous or non-synonymous variants. The former indicates that the same amino acid is encoded when polymorphism occurs, however, the latter means that the encoded amino acid may be changed in the related protein thereby exerting an impact on diseases' development including cancer 19, 21-23. Some SNPs within lncRNA genes have been demonstrated to affect the expression and action of lncRNAs, which are identified as regulatory RNAs without protein-coding potential, and then affect individual cancer susceptibility and patient survival 24, 25.

Uterine cervical cancer is known as second common type of gynecological cancer in Taiwan. To our knowledge, no investigation has associated GAS5 genetic variants with cervical cancer in Taiwanese women. Therefore, we designed this study to explore the relationships of GAS5 genetic variants with the development of cervical cancer and patient survival.

## Materials and Methods

### Enrolled participants

The study was designed to explore the relationships of GAS5 genetic variants to the development of uterine cervical cancer and patient survival retrospectively. Two hundred and eight Taiwanese patients having uterine cervical neoplasia were recruited, including 111 women with invasive cancer and 97 with precancerous lesions based on pathologic report, who received treatment at the Department of Obstetrics and Gynecology in Chung Shan Medical University Hospital in Taichung, Taiwan from May 1994 to April 2015. Three hundred and seven normal women, who received general examination at the Outpatient Patient Department, were enrolled as controls in this period as well. Cervical neopolasia patients underwent standard treatment protocols in this hospital. Normal cytologic results were reported for the control women and further defined based on detailed colposcopic findings. Chung Shan Medical University Hospital institutional review board agreed with this study (CSMUH No: CS18208).

### Extracted deoxyribonucleic acid (DNA) from blood specimens of all participants for the distribution of GAS5 genetic variants

The laboratory technicians got the blood samples from all recruited women by venipuncture. These specimens were then mingled with ethylenediaminetetraacetic acid collected in Vacutainer tubes. The blood samples were soon stored at 4°C. The staffs subsequently extracted DNA from leukocytes in compliance with previously described publication and then dissolved the extracts into pH 7.8 TE buffer 26, 27. The products were finally stored at -20°C and were regarded as templates for the polymerase chain reaction (PCR).

### Selection of GAS5 genetic polymorphisms

Two GAS5 genetic variants rs145204276 and rs55829688 were detected in compliance with the data of International HapMap Project and previous study 28. The polymorphism rs145204276 AGGCA/- was demonstrated to be located at the promoter region of lncRNA GAS5 and was associated with the progression of several cancers 28, 29. GAS5 genetic polymorphisms rs145204276 and rs55829688 were examined by ABI StepOne Real-Time PCR System (Applied Biosystems, Foster City, CA, USA) as well as delineated with SDS vers. 3.0 software, as our previous publication.

### Genotyping of GAS5 polymorphisms

Genotypes of lncRNAs GAS5 genetic variants rs145204276 and rs55829688 were determined by ABI StepOne Real-Time PCR System (Applied Biosystems, Foster City, CA, USA), and analyzed with SDS vers. 3.0 software, as described previously 29.

### Statistical analysis

Post hoc analysis was performed by Scheffe test after analysis of variance (ANOVA) was used to compare the age distribution in the enrolled participants. Hardy-Weinberg equilibrium was applied to check the genotypic distributions of rs145204276 and rs55829688 in control women [degree of freedom (d.f.)=2]. Chi-square or Fisher exact tests were applied to examine the relationships of two GAS5 genetic variants with the cervical tumorigenesis. Logistic or multinomial logistic regression models were applied to acquire adjusted odds ratios (AORs) and their 95% confidence intervals (95% CIs) after age adjustment. Above methods were also performed to associate the distributions of two GAS5 polymorphisms with a variety of clinicopathological factors.

The influences of GAS5 genetic variants and clinicopathological parameters on 5 years survival of patients with invasive cervical cancer were plotted using Kaplan-Meier curves for univariate analysis. The log-rank test was done to check the differences among them. The impacts of GAS5 polymorphisms and these clinicopathological variables on 5 years survival of these patients were checked using Cox proportional hazard model for multivariate analysis in relation to survival time. The SPSS, version 12.0 and WinPepi Software, version 10.0 were performed for analyzing statistical significance. Hazard ratios (HRs) and their 95% confidence intervals (CIs) were determined by the SPSS, version 12.0. *P* <0.05 was identified to exhibit a significant difference.

## Results

The age was differently distributed with a statistical significance between patients with cervical neoplasia and normal control women (49.8 ± 13.5 vs. 43.8 ± 10.1, *p*<0.001). Using ANOVA with Scheffe test for post hoc analysis, the age was differently distributed between patients with cervical invasive cancer and those with precancerous lesion (54.4 ± 12.3 vs. 44.5 ± 12.9, *p*<0.001), and between those with cervical invasive cancer and control women (54.4 ± 12.3 vs. 43.8 ± 10.1, *p*<0.001). But, there was no significant difference between patients with precancerous lesions and control women (44.5 ± 12.9 vs. 43.8 ± 10.1, *p*= 0.862). The minor allele frequency (MAF) of GAS5 genetic variant rs145204276 accorded with the Hardy-Weinberg equilibrium [χ2 value, 1.172, *p*=0.556; d.f.=2]. It was calculated based on normal control. The MAF of GAS5 rs55829688 was also in line with the Hardy-Weinberg equilibrium [χ2 value, 0.0389, *p*=0.981; d.f.=2].

The genetic distribution of GAS5 rs145204276 polymorphism exerted no significance difference between patients with cervical neoplasia and control women (*p*=0.241; Table 1). It still revealed no significant difference between these subjects after age adjustment (*p*=0.236; Table 1). When AGGCA/AGGCA (insertion/insertion; Ins/Ins) was used as a reference, AGGCA/deletion (AGGCA/-, Ins/Del) and Del/Del (-/-) could not exhibit different genotypic frequency between patients with cervical neoplasia and normal women in GAS5 rs145204276 polymorphism (*p*= 0.690, AOR=0.93, 95% CI=0.64-1.34; Table 1). While Ins/Ins and Ins/Del were used as references, Del/Del also presented no difference between them (*p*= 0.091, AOR=0.62, 95% CI=0.36-1.08; Table 1). Meanwhile, neither was significant differences demonstrated in GAS5 rs55829688 between the patients with cervical neoplasia and the normal controls, nor was statistical difference noted after age adjustment in this genetic polymorphism (Table 1).

If patients having cervical neoplasia group was subdivided into those with invasive cancer and precancerous subgroups, it revealed no significant difference in the genotypic frequencies of Ins/Ins, Ins/Del and Del/Del in GAS5 rs145204276 among patients with invasive cancer and those with precancerous lesions of uterine cervix as well as control women (*p* = 0.144; Table 2). While Ins/Inn and Ins/Del were used as references, Taiwanese women with precancerous lesions had significantly lower rate of genotype Del/Del (*p*= 0.030, AOR=0.40, 95% CI=0.18-0.92; Table 2). However, there was no different Del/Del distribution between patients with invasive cancer and normal controls (*p*= 0.624). There were also no different distributions of Ins/Del and Del/Del among these subjects while Ins/Ins was used as a reference. Meanwhile, there were no different genotypic frequencies between patients with cervical invasive cancer and normal controls as well as between patients with precancerous lesions and normal controls after age adjustment in GAS5 rs55829688 (Table 2).

While associating GAS5 genetic variants with clinicopathological factors of cervical cancer, it revealed no significant associations of rs145204276 with these factors (Table 3). In addition, GAS5 rs55829688 also could not exerted significant associations with clinicopathological characteristics (data not shown). When the distributions of GAS5 genetic polymorphism were analyzed among all cancer stage subdivisions (stage I, II, III and IV) in patients with uterine cervical cancer, we found that patients with genotype TC in GAS5 rs55829688 had more risk to have stage II (OR: 2.69, 95% CI: 1.08-6.70; *p*=0.033) and stage III (OR: 15.56, 95% CI: 1.80-134.24; *p*=0.013) using TT as a reference (Supplement Table 1). In addition, cervical cancer patients with genotypes TC/CC in rs55829688 had more risk to have stage III (OR: 12.73, 95% CI: 1.49-108.84; *p*=0.020). However, the sample numbers were obviously limited.

Cervical cancer patients with Ins/Del and Del/Del presented 5 years survival rate 0.79 (95% CI=0.68-0.89), as compared to those with Ins/Ins 0.86 (95% CI=0.76-0.97) in GAS5 rs145204276 (Table 4). Patient with Del/Del had 5 years survival rate 0.94 (0.82-1.00), while compared to those with Ins/Ins and Ins/Del having 5 years survival rate 0.80 (95% CI=0.71-0.88). However, both above results could not reach significant differences (data not shown). In the meantime, cervical cancer patients with TC/CC had 5 years survival rate 0.81 (95% CI=0.69-0.92), in comparison with those having TT 0.81 (95% CI=0.70-0.92) in GAS5 rs55829688. Patient with C/C had 5 years survival rate 0.80 (95% CI=0.45-1.00), while compared to those with TT/TC exhibiting 5 years survival rate 0.81 (95% CI=0.73-0.89). Among those clinicopathological factors, cervical patients with positive pelvic lymph nodes presented most obviously poor 5 years survival rate 0.52 (95% CI=0.32-0.72), as compared to those with negative pelvic lymph nodes having 5 years survival rate 0.92 (95% CI=0.86-0.98; Table 4).

**Figure 1:**
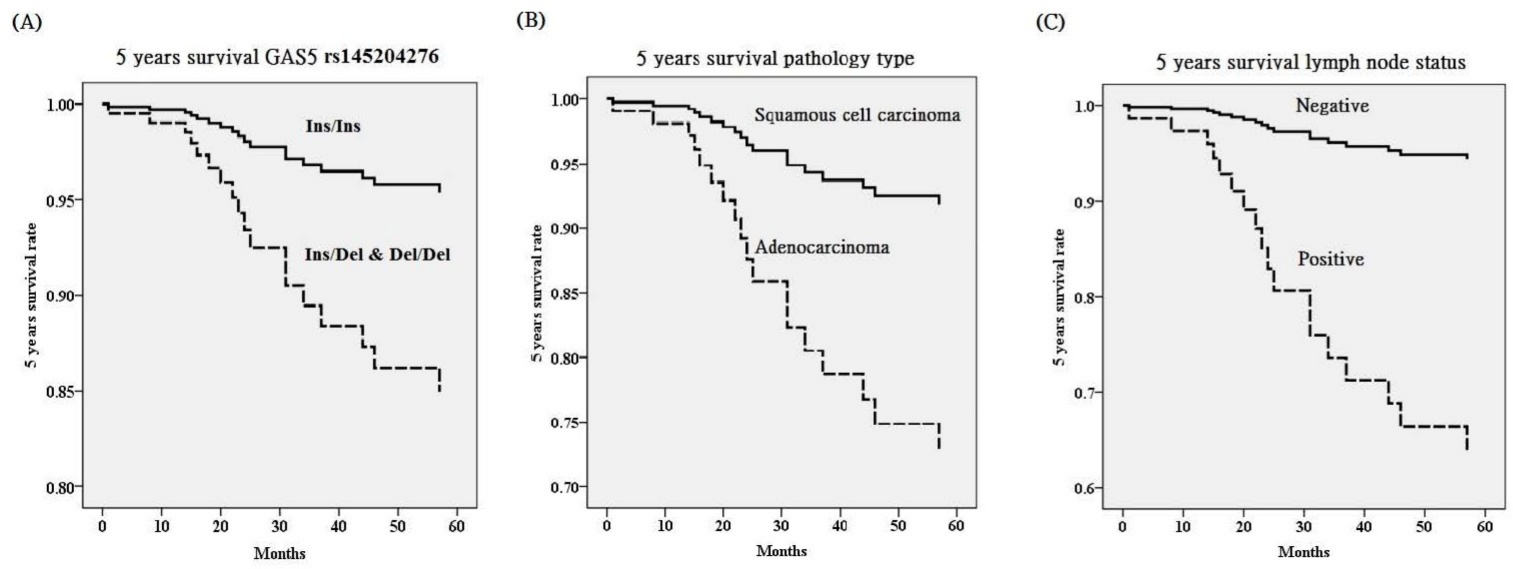
Five years survival rate based on polymorphisms of long noncoding RNA growth arrest-specific transcript 5 (GAS5) (A), pathologic type (B) and lymph node status (C) using Cox proportional hazard model after adjusting for various GAS5 polymorphisms and clinicopathological characteristics. Cervical cancer patients with Ins (insertion, AGGCA)/Del (deletion) and Del/Del (-/-) in GAS5 rs145204276 [hazard ratio (HR): 3.45, 95% confidence (95% CI): 1.01-11.82; *p*=0.049], adenocarcinoma (HR: 3.71, 95%CI: 1.02-13.49; *p*=0.046) and positive lymph node metastasis (HR: 7.78, 95%CI: 1.85-32.62; *p*=0.005) have poorer 5 years survival HR.

Genotypic distribution of GAS5 rs145204276 for cervical cancer patients revealed that patients with Ins/Del and Del/Del tended to have poorer 5 years survival HR, compared to those with Ins/Ins after adjusting GAS5 polymorphisms and various clinicopathological factors (*p*=0.049, HR=3.45, 95% CI=1.01-11.82; Table 5, Figure 1). However, patients with Del/Del could not present this tendency as compared to those with Ins/Ins and Ins/Del in GAS5 rs145204276. GAS5 rs55829688 also could not exhibit the tendency. Cervical cancer patients with adenocarcinoma pathologic type tended to have poorer 5 years survival HR, compared to those with squamous cell pathologic type (*p*=0.046, HR=3.71, 95% CI=1.02-13.49; Table 5). Furthermore, positive pelvic lymph node metastasis predicted most obviously poor 5 years survival HR after adjusting two GAS5 genetic variants and various clinicopathological parameters (*p*=0.005, HR=7.78, 95% CI=1.85-32.62; Table 5).

## Discussion

The study explores that Taiwanese women with precancerous lesions had significantly lower rate of genotype Del/Del in GAS 5 rs145204276. But women with cervical invasive cancer could not present different Del/Del rate. The GAS5 is known as a tumor suppressor in human cancer. It has been demonstrated that GAS5 is down-regulated in many cancers, such as breast cancer, hepatocellular carcinoma, lung cancer and ovarian cancer 14-18. The patients with precancerous lesions had higher rate of Ins/Ins and Ins/Del in GAS5 rs145204276. The function of GAS5 may be affected by the genetic variants but still preserves tumor suppression function. Therefore, it is suggested that the effect of association of Del/Del with precancerous lesions is not strong enough to destroy the basement membrane of cervical epithelium and induce invasiveness of cervical cancer cells. There were no significant genotypic distributions among patients with cervical invasive cancer and those with precancerous lesions as well as normal controls in GAS5 rs55829688. To the best of our knowledge, few investigations reported the implication of GAS5 polymorphisms in cervical cancer. However, Yang et al. demonstrated that elevated expression of GAS5 might reduce cell proliferation of cervical cancer via down-regulating miR-196a and miR-205 30.

As compared to controls, osteosarcoma patients were demonstrated to display significantly lower frequency of genotype Del/Del in GAS5 rs145204276 by Xu et al. 31. Patients with Del/Del exhibited significantly elevated expression of GAS5 as compared to those with Ins/Ins. GAS5 rs145204276 is located at promoter area and may modulate GAS5 expression by affecting methylation status at the 7th CpG site 31-33. It seems reasonable because increased GAS5 expression has been reported to inhibit cell growth of osteosarcoma through the mIR-221/aplasia Ras homologue member I pathway 8. It was also revealed that the rate of genotype Del/Del in GAS5 rs145204276 was statistically lower in patients with gastric cancer than in the controls by Li et al. 34. They demonstrated if gastric cancer patients had allele deletion they displayed higher GAS5 expression in their cancer tissue samples. They further found that patients with Del/Del exhibited higher methylation percentage in the 7th CpG. Although hypermethylation is usually related to lower transcriptional activity and reduces gene expression, it occasionally probably leads to the shift of transcription start site from one to another, and then induces active transcription. With this hypermethylation model, Del/Del was found to activate transcription and subsequently elevate GAS5 expression in their study 34. In addition, GAS5 is widely regarded as a tumor suppressor gene because down-regulation of GAS5 has been demonstrated in a variety of cancer 7, 35, 36. Tang et al. demonstrated that deletion allele in GAS5 rs145204276 might protect against the susceptibility to breast cancer via the induction of promoter activity by binding to transcriptional factor specificity protein 1, and subsequently led to elevated GAS5 expression 37. However, Zheng et al. revealed that both the genotypes Ins/Del and Del/Del exhibited reduced susceptibility to colorectal cancer 38. In contrast to above findings, Tao et al. demonstrated that the allele deletion in rs145204276, which is 5-bp indel polymorphism shown as -/AGGCA as well as -/- and is situated at the promoter area, is correlated with methylation, and then exhibited higher GAS5 expression in hepatocellular carcinoma (HCC) 32. Elevated GAS5 might present as a proto-oncogene in HCC, in contrast with its inhibitory role in other cancers, and subsequently could elevate the susceptibility to HCC.

Thereafter, we investigated the associations of GAS5 genetic variants with various clinicopathological factors of uterine cervical cancer. The statistical analysis could not reveal significant association among GAS5 polymorphisms rs145204276 and rs55829688 and these parameters. As we know, no study reported the association of GAS5 genetic variants with clinicopathological factors of cervical cancer in Taiwan. When the distributions of GAS5 genetic polymorphism were analyzed among all cancer stage subdivisions (stage I, II, III and IV) in patients with uterine cervical cancer, we found that patients with genotype TC or TC/CC in GAS5 rs55829688 have more risk to have stage II or stage III using TT as a reference. However, the sample numbers were obviously limited. Large sample size is necessary in the future investigation. Considering the gene expression of GAS5, Wang et al. found that the GAS5 expression was statistically related to stage and lymph node metastasis of cervical cancer 39. Furthermore, Li et al. revealed that the expression of GAS5 was significantly associated with stage and lymph node metastasis of cervical cancer but not associated with tumor size and pathologic type 40. However in gastric cancer, Li et al. revealed that gastric cancer patients with late tumor stage were found to exhibit a significantly lower rate of Del/Del in GAS5 rs145204276 than those with early tumor stage 34. Zheng et al. revealed that colorectal cancer patients with allele deletion in GAS5 rs145204276 had less risk to develop lymph node metastasis 38.

This study revealed that cervical patients with Ins/Del and Del/Del in rs145204276 tended to have poorer 5 years survival HR, compared to those with Ins/Ins after adjusting GAS5 polymorphisms and various clinicopathological factors. However, there is a paucity of knowledge regarding the impact of GAS5 genetic variants on cervical cancer patient survival in Taiwan. It was however revealed that gastric cancer patients with genotype Del/Del had displayed better overall survival rate 34. Sun et al. demonstrated that patients with gastric cancer, who exhibited low GAS5 expression, had a tendency to have poor disease-free survival 18. This proposed that allele deletion in GAS5 rs145204276 exerted a protective action in patients with gastric cancer through the modulation of GAS5 transcript. In contrast, Liu et al. inferred that GAS5 was displayed as a competing endogenous RNA and served a role in the pathogenesis of gastric cancer 41. However, pelvic lymph node status rather than GAS5 genetic variants was the most significantly predictable parameter of 5 years survival HR in Taiwanese patients with cervical cancer among GAS5 polymorphisms and a variety of clinicopathological factors in this study 42, 43.

This investigation has two main limitations. First, the sample size of the cervical cancer patients who were enrolled in this investigation was relatively small. Therefore, statistical analysis of the implication of GAS5 polymorphism in patient survival was not significant enough to draw a definite association. Concerned with the relationships of allele deletion in GAS5 rs145204276 with cancer development and progression, there were diverse results in previous studies 31-34, 37. The mechanism delineating the impact of rs145204276 on methylation status of the GAS5 promoter and transcription activity as well as subsequent GAS5 expression needs further investigation to clarify the exact role of GAS5 polymorphisms in uterine cervical cancer.

In conclusion, patients with precancerous lesion of uterine cervix exhibited lower rate of Del/Del in GAS5 rs145204276 but those with invasive cancer could not present the finding. Cervical cancer patients with allele deletion in rs145204276 had the tendency to present poorer HR of 5 years survival rate. There were no associations of GAS5 rs55829688 with development of cervical cancer and patient survival. However, mechanism delineating the influence of allele deletion in rs145204276 on GAS5 promoter methylation and thereafter on transcription action as well as subsequent GAS5 expression needs further investigation to elucidate the exact role of GAS5 genetic variant in uterine cervical cancer in the near future.

## Supplementary Material

Supplementary figures and tables.

## Figures and Tables

**Table 1:** Distributions of genetic variant of the long noncoding RNA growth arrest-specific transcript 5 (GAS5) in Taiwanese women who had uterine cervical neoplasias or control women.

GAS5 genetic polymorphisms	Control women (n= 307, %)	Cervical neoplasias^a^ (n= 208, %)	ORs (95% CIs)	*p* values	AORs (95% CIs)^b^	Adjusted *p* values^b^
rs145204276						
Ins/Ins^c^	119, 38.8 %	83, 39.9%	1.00	0.241	1.00	0.236
Ins/Del	138, 45.0 %	102, 49.0%	1.06 (0.73-1.55)	0.765	1.03 (0.70-1.53)	0.873
Del/Del	50, 16.3 %	23, 11.1%	0.66 (0.37-1.16)	0.151	0.63 (0.35-1.14)	0.129
Ins/Ins^c^	119, 38.8 %	83, 39.9%	1.00	0.795	1.00	0.690
Ins/Del & Del/Del	188, 61.2%	125, 60.1%	0.95 (0.67-1.37)		0.93 (0.64-1.34)	
Ins/Ins & Ins/Del^c^	257, 83.7%	185, 88.9%	1.00	0.097	1.00	0.091
Del/Del	50 16.3%	23, 11.1%	0.64 (0.38-1.08)		0.62 (0.36-1.08)	
rs55829688						
TT^c^	148, 50.2%	99, 50.0%	1.00	0.632	1.00	0.880
TC	121, 41.0%	86, 43.4%	1.06 (0.73-1.55)	0.752	1.02 (0.69-1.50)	0.932
CC	26, 8.8%	13, 6.6%	0.75 (0.37-1.53)	0.423	0.84 (0.40-1.75)	0.643
TT^c^	148, 50.2%	99, 50.0%	1.00	0.971	1.00	0.949
TC/CC	147, 49.8%	99, 50.0%	1.01 (0.70-1.44)		0.99 (0.68-1.43)	
TT/TC^c^	269, 91.2%	185, 93.4%	1.00	0.366	1.00	0.619
CC	26, 8.8%	13, 6.6%	0.73 (0.36-1.45)		0.84 (0.41-1.70)	

Statistical analysis: chi-square test or logistic regression model. ^a^Cervical neoplasias included precancerous lesions and invasive cancer ^b^Adjusted *p* values as well as AORs and their 95% CIs were checked by logistic regression model after adjusting age. ^c^As a reference for comparison to define the odds ratios of other genetic variants. 95% CIs, 95% confidence intervals; AORs, adjusted odds ratios; Ins, insertion, presence of AGGCA; Del, deletion, absence of AGGCA.

**Table 2:** Genetic variant distributions of the long noncoding RNA growth arrest-specific transcript 5 (GAS5) in Taiwanese women with uterine cervical invasive cancer or precancerous lesions and normal controls.

GAS5 genetic polymorphisms	Normal controls (n=307, %)	Pre- cancerous lesions (n =97, %)	Invasive cancer (n=111, %)	*p* values	AORs (95% CIs)^a^	Ad. *p* values	AORs (95% CIs)^b^	Ad. *p* values
rs145204276								
Ins/Ins^c^	119, 38.8 %	36, 37.1%	47, 42.3%	0.144	1.00		1.00	
Ins/Del	138, 45.0 %	54, 55.7%	48, 43.2%		1.29 (0.79-2.10)	0.308	0.80 (0.48-1.33)	0.387
Del/Del	50, 16.3 %	7, 7.2%	16, 14.4%		0.46 (0.19-1.11)	0.085	0.75 (0.37-1.54)	0.436
Ins/Ins^c^	119, 38.8 %	36, 37.1%	47, 42.3%	0.718	1.00		1.00	
Ins/Del & Del/Del	188, 61.2%	61, 62.9%	64, 57.7%		1.07 (0.67-1.71)	0.781	0.79 (0.49-1.26)	0.321
Ins/Ins & Ins/Del^c^	257, 83.7%	90, 92.8%	95, 85.6%	0.082	1.00		1.00	
Del/Del	50 16.3%	7, 7.2%	16, 14.4%		0.40 (0.18-0.92)	0.030	0.85 (0.43-1.65)	0.624
rs55829688								
TT^c^	148, 50.2%	45, 48.4%	54, 51.4%	0.747	1.00		1.00	
TC	121, 41.0%	40, 43.0%	46, 43.8%		1.08 (0.66-1.77)	0.752	0.97 (0.59-1.59)	0.896
CC	26, 8.8%	8, 8.6%	5, 4.8%		1.02 (0.43-2.41)	0.964	0.63 (0.22-1.82)	0.393
TT^c^	148, 50.2%	45, 48.4%	54, 51.45	0.912	1.00		1.00	
TC/CC	147, 49.8%	48, 51.6%	51, 48.6%		1.07 (0.67-1.71)	0.773	0.92 (0.57-1.48)	0.719
TT/TC^c^	269, 91.2%	85, 91.4%	100, 95.2%	0.402	1.00		1.00	
CC	26, 8.8%	8, 8.6%	5, 4.8%		0.98 (0.43-2.26)	0.970	0.64 (0.23-1.81)	0.398

^a^Adjusted *p* values and AORs with their 95% CIs were defined using multinomial logistic regression model after controlling age between patients with cervical precancerous lesions and control women. ^b^Adjusted *p* values and AORs with their 95% CIs were defined using multinomial logistic regression model after controlling age between patients with cervical invasive cancer and control women. ^c^Used as a reference for comparison to define the odds ratios of other genotypes. AORs, adjusted odds ratios; 95% CIs, 95% confidence intervals; Ad. *p*, adjusted *p*; Ins, insertion, presence of AGGCA; Del, deletion, absence of AGGCA.

**Table 3:** Associations of genetic variant distribution of long noncoding RNA growth arrest-specific transcript 5 (GAS5) with clinicopathological factors of the patients with uterine cervical invasive cancer.

	GAS5 rs145204276			
Variables^a^	Ins/Ins Ins/del^b^	Del/del	*p* value	ORs (95% CIs)
Clinical stage N (%)			0.332	
stage I^b^	53 (82.8)	11 (17.2)		1.00
≥ stage II	42 (89.4)	5 (10.6)		0.57 (0.19-1.78)
Pathologic type N (%)			0.683	
squamous cell carcinoma^b^	85 (85.9)	14 (14.1)		1.00
adenocarcinoma	10 (83.3)	2 (16.7)		1.21 (0.24-6.14)
Cell grading N (%)			0.226	
well (grade 1)^b^	11 (73.3)	4 (26.7)		1.00
moderate & poor (grades 2/3)	84 (87.5)	12 (12.5)		0.39 (0.11-1.43)
Stromal invasion depth N (%)			0.550	
≤10 mm^b^	49 (83.1)	10 (16.9)		1.00
>10 mm	41 (87.2)	6 (12.8)		0.72 (0.24-2.14)
Tumor diameter^b^ N (%)			0.231	
≤ 4cm	50 (82.0)	11 (18.0)		1.00
>4cm	45 (90.0)	5 (10.0)		0.51 (0.16-1.57)
Parametrium N (%)			0.089	
no invasion^b^	56 (81.2)	13 (18.8)		1.00
invasion	39 (92.9)	3 (7.1)		0.33 (0.09-1.24)
Vagina N (%)			0.610	
no invasion^b^	59 (84.3)	11 (15.7)		1.00
invasion	36 (87.8)	5 (12.2)		0.75 (0.24-2.32)
Pelvic lymph node N (%)			0.757	
no metastasis^b^	71 (84.5)	13 (15.5)		1.00
metastasis	24 (88.9)	3 (11.1)		0.68 (0.18-2.60)

Statistical analyses: chi-square or Fisher's exact tests ^a^Some clinicopathological data could not be obtained from the patients with cervical invasive cancer due to incomplete medical charts or records. ^b^As a reference. ORs, odds ratios; 95% CIs, 95% confidence intervals; Ins, insertion, presence of AGGCA; Del, deletion, absence of AGGCA.

**Table 4:** Five years survival rate based on genetic variants of long noncoding RNA growth arrest-specific transcript 5 (GAS5) and various clinicopatholgical characteristics in uterine cervical cancer patients.

Characteristics	5 years survival rate & 95% CI
GAS5	
rs145204276	
Ins/Del & Del/Del vs Ins/Ins^a^	0.79 (0.68-0.89) vs 0.86 (0.76-0.97)
Del/Del vs Ins/Ins & Ins/Del^a^	0.94 (0.82-1.00) vs 0.80 (0.71-0.88)
rs55829688	
TC/CC vs TT^a^	0.81 (0.69-0.92) vs 0.81 (0.70-0.92)
CC vs TT/TC^a^	0.80 (0.45-1.00) vs 0.81 (0.73-0.89)
Clinical stage	
≥ stage II vs stage I^a^	0.72 (0.59-0.86) vs 0.90 (0.82-0.97)
Pathologic type	
adenocarcinoma vs squamous cell carcinoma^a^	0.68 (0.43-0.94) vs 0.84 (0.76-0.92)
Cell grading	
moderate & poor (grades 2/3) vs well (grade 1)^a^	0.82 (0.74-0.90) vs 0.81 (0.62-1.00)
Stromal invasion depth	
>10 mm vs ≤10 mm^a^	0.69 (0.55-0.82) vs 0.92 (0.85-1.00)
Tumor diameter	
>4 cm vs ≤ 4cm^a^	0.71 (0.58-0.85) vs 0.91 (0.83-0.99)
Parametrium	
invasion vs no invasion^a^	0.71 (0.56-0.85) vs 0.89 (0.81-0.97)
Vagina	
invasion vs no invasion^a^	0.73 (0.58-0.88) vs 0.87 (0.78-0.95)
Pelvic lymph node	
metastasis vs no metastasis^a^	0.52 (0.32-0.72) vs 0.92 (0.86-0.98)

Statistical analyses: Kaplan-Meier curve model ^a^As a comparison reference 95% CI, 95% confidence interval; Ins, insertion, presence of AGGCA; Del, deletion, absence of AGGCA.

**Table 5:** Impact of genetic variants of and various clinicopatholgical characteristics on the 5 years survival rate in uterine cervical cancer patients.

Characteristics	5 years survival HR	
	*p* value	HR & 95% CI^b^
GAS5		
rs145204276		
Ins/Del & Del/Del vs Ins/Ins^a^	0.049	3.45 (1.01-11.82)
Del/Del vs Ins/Ins & Ins/Del^a^	0.162	0.21 (0.02-1.88)
rs55829688		
TC/CC vs TT^a^	0.431	0.63 (0.20-1.97)
CC vs TT/TC^a^	0.214	5.56 (0.37-82.87)
Pathologic type		
adenocarcinoma vs squamous cell carcinoma^a^	0.046	3.71 (1.02-13.49)
Pelvic lymph node		
metastasis vs no metastasis^a^	0.005	7.78 (1.85-32.62)

Statistical analyses: Cox proportional hazard model ^a^As a comparison reference HR, hazard ratio and 95% CI, 95% confidence interval for GAS5 genetic variants and clinicopathological characteristics, compared with their respective controls. Ins, insertion, presence of AGGCA; Del, deletion, absence of AGGCA.
