# Supplementary material for: The relationships of genetic polymorphisms of the long noncoding RNA growth arrest-specific transcript 5 with uterine cervical cancer
Source: Int J Med Sci. 2020 May 18;17(9):1187–95. doi: 10.7150/ijms.44583 (PMC7294910; doi:10.7150/ijms.44583)
Supplement: Supplementary file 1 — Supplementary figures and tables. [file ijmsv17p1187s1.pdf]

Supplement Table 1. The distributions of GAS5 genetic polymorphism among all cancer stage subdivisions (stage I, II, III and IV) in patients with uterine cervical cancer

GAS5 genetic polymorphisms	Stage I[a]	Stage II	Stage III	Stage IV	*p* value
rs145204276					0.810
Ins/Ins[b]	27	12	5	3	
Ins/Del	24	17	4	3	
Del/Del	11	4	0	1	
OR (95% CI) Ins/Del vs Ins/Ins[b]	1.00	1.59 (0.63-4.00)	0.90 (0.22-3.74)	1.13 (0.21-6.11)	
& *p* value		0.321	0.885	0.891	
OR (95% CI) Del/Del vs Ins/Ins[b]	1.00	0.82 (0.22-3.10)	u.a	0.82 (0.08-8.75)	
& *p* value		0.768	u.a	0.868	
Ins/Ins[b]	27	12	5	3	0.746
Ins/Del & Del/Del	35	21	4	4	
OR (95% CI) Ins/Del & Del/Del vs Ins/Ins[b]	1.00	1.35 (0.57-3.22)	0.62 (0.15-2.52)	1.03 (0.21-4.99)	
& *p* value		0.498	0.501	0.972	
Ins/Ins & Ins/Del[b]	51	29	9	6	0.614
Del/Del	11	4	0	1	
OR (95% CI) Del/Del vs Ins/Ins & Ins/Del[b]	1.00	0.64 (0.19-2.19)	u.a.	0.77 (0.08-7.08)	
& *p* value		0.477	u.a.	0.820	
rs55829688					0.022
TT[b]	35	13	1	5	
TC	18	18	8	2	
CC	4	1	0	0	
OR (95% CI) TC vs TT[b]	1.00	2.69 (1.08-6.70)	15.56 (1.80-134.24)	0.78 (0.14-4.41)	
& *p* value		0.033 *	0.013 *	0.777	
OR (95% CI) CC vs TT[b]	1.00	0.67 (0.07-6.59)	u.a.	u.a.	
& *p* value		0.734	u.a.	u.a.	
TT[b]	35	13	1	5	0.011
TC/CC	22	19	8	2	
OR (95% CI) TC/CC vs TT[b]	1.00	2.33 (0.96-5.63)	12.73 (1.49-108.84)	0.64 (0.11-3.57)	
& *p* value		0.061	0.020 *	0.607	
TT/TC[b]	53	31	9	7	0.850
CC	4	1	0	0	
OR (95% CI) CC vs TT/TC[b]	1.00	0.43 (0.05-4.00)	u.a.	u.a.	

& *p* value	0.456	u.a.	u.a.

Statistical anslysis Fisher's exact test and multinomial logistic regression model

[*] $P<0.05$

[a]Used as a reference for comparison to define the odds ratios of other stages.
[b]Used as a reference for comparison to define the odds ratios of other genotypes.
GAS5, growth arrest-specific transcript; OR, odds ratio; 95% CI, 95% confidence interval; u.a., unavailable
